# Editorial: Wine Microbiology: Current Trends and Approaches

**DOI:** 10.3389/fmicb.2022.873980

**Published:** 2022-03-29

**Authors:** Antonio Bevilacqua, Leonardo Petruzzi, Maria Arevalo-Villena, Panagiotis Kandylis, Aspasia Nisiotou

**Affiliations:** ^1^Department of Agriculture, Food, Natural Resources and Engineering, University of Foggia, Foggia, Italy; ^2^Analytical Chemistry and Food Technology Department, Faculty of Chemical Sciences and Technologies, Castilla-La Mancha University, Ciudad Real, Spain; ^3^School of Agriculture, Faculty of Agriculture, Forestry and Natural Environment, Aristotle University of Thessaloniki, Thessaloniki, Greece; ^4^Hellenic Agricultural Organization “Dimitra”, Institute of Technology of Agricultural Products, Lykovrysi, Greece

**Keywords:** wine, microbiology, *Saccharomyces*, non-*Saccharomyces*, microbiota diversity

Wine has long been characterized as a product that conveys significant socio-economic impact. Its production involves complicated biochemical reactions, carried out through the presence of complex microbial communities. Although *Saccharomyces cerevisiae* has been the most utilized species, mixed cultures with other *Saccharomyces* and/or non-*Saccharomyces* species have shown to be useful and sometimes preferred in fermentation trials. Many researchers support the idea of preserving the autochthonous microbiota, which can be associated with a distinct terroir; therefore, the concept of “microbial terroir” is considered to be emerging as a principal element in modern winemaking. With an increasing understanding of microbial diversity and its effects on wine fermentation, wine production can be optimized by enhancing the expression of regional characteristics by understanding and managing the microbes present.

Other Topics worthy of investigation for wine microbiology are the validation of standard protocols for a robust characterization of starter cultures, as well as the use of immobilized microorganisms and/or enzymes to improve wine aroma. All these subjects are covered in this Research Topic by an international team composed of researchers from Argentina, Cyprus, Greece, Italy, Spain, Switzerland, and the United States of America covering different areas of expertise and research lines (statistic and method validation, characterization of autochthonous microbiota, microbiology in the context of climate changes, enzyme and yeast immobilization, and metataxonomic analysis).

Romano et al. proposed a protocol for the characterization of wine strains of *S. cerevisiae*. The authors carried out inter-laboratory-scale comparative fermentations performed by 17 Research Units of the Italian Group of Microbiology of Vine and Wine (GMVV) using two commercial *S. cerevisiae* strains, both in synthetic medium and grape musts. The paper reports on reproducible, replicable, and statistically valid results thus reducing variability amongst independent batches to acceptable levels.

Vaquero et al. presented research on ternary fermentations in which *Lachancea thermotolerans* had been co-inoculated with *Hanseniaspora vineae, Torulaspora delbrueckii*, or *Metschnikowia pulcherrima*, and the fermentation process was subsequently completed with sequential inoculation of *S. cerevisiae*. The authors recorded significant inhibition of lactic acid production in mixed fermentations of *L. thermotolerans* with *H. vineae*. On the other hand, the presence of *M. pulcherrima* boosted lactic acid production by *L. thermotolerans*. Good consortia strategies are pivotal for producing more complex wines, especially in warm regions where quality-related compounds undergo a marked degradation.

Considering the interest in stable/immobilized enzymes for winemaking, Fernández-Pacheco et al. evaluated the effect of free and immobilized commercial β-glucosidases on aroma compounds in white wines. In their Research, enzyme immobilization contributed to a high concentration of some volatile compounds such as nerol or geraniol.

Chalvantzi et al. focused on the culturable fraction of the vineyard-associated yeast biota in Greece. The authors observed defined structures in the wine yeast communities both at vineyard and regional scales. Key wine yeast populations had a substantial role in region delimitation, showing their potential effect on wine regionality. Environmental factors could only moderately explain community structure, with maximum temperature, elevation, and net precipitation showing the highest correlation with the yeast community patterns, while certain factors could be associated with certain yeast populations.

Rivas et al. studied the influence of the challenging climatic conditions encountered in Argentina on the bacterial diversity in soil and wine for 3 consecutive years. Although a stable core of bacterial phyla in both soil and wine was recorded across vintages, the authors recorded changes in the bacterial diversity as a response to harsh environmental events.

Kamilari et al. assessed the existence of different microbial patterns among grapes growing in geographically distant areas of Cyprus. The authors identified microbial biomarkers linked to each geographical area and grape variety.

Finally, Griggs et al. reviewed factors contributing to inter-annually stable seeding events to initiate microbiome re-assembly on grapevines seasonally, including cultivar, phenology, weather, and viticultural management practices. The authors concluded that soil and landscape attributes, like plant life, constitute a local pool of microbial diversity, while regional weather patterns facilitate microbial dispersion. The origin and the stability of microbiota were characterized as inter-annual features of specific vineyards.

All articles of this Research Topic open a window on the new frontiers of wine microbiology and on the challenges that microbiologists all over the world have to contend with. We hope that readers could find in this Research Topic of Articles new details and information to expand their knowledge and increase their curiosity.

## Author Contributions

All authors listed have made a substantial, direct, and intellectual contribution to the work and approved it for publication.

## Conflict of Interest

The authors declare that the research was conducted in the absence of any commercial or financial relationships that could be construed as a potential conflict of interest.

## Publisher's Note

All claims expressed in this article are solely those of the authors and do not necessarily represent those of their affiliated organizations, or those of the publisher, the editors and the reviewers. Any product that may be evaluated in this article, or claim that may be made by its manufacturer, is not guaranteed or endorsed by the publisher.

